# Can remimazolam be safely used in cardiac tamponade?: A case report

**DOI:** 10.1097/MD.0000000000034822

**Published:** 2023-08-11

**Authors:** Hyun Joo Heo, Geonbo Kim, Yu Yil Kim, Junyoung Park

**Affiliations:** a Department of Anesthesiology and Pain Medicine, Presbyterian Medical Center, Jeonju, Jeollabuk-Do, Korea.

**Keywords:** cardiac tamponade, case report, general anesthesia, hemodynamic instability, remimazolam

## Abstract

Remimazolam, a benzodiazepine drug, has recently been developed and is currently used for the induction and maintenance of anesthesia. Remimazolam provides hemodynamic stability during anesthesia induction. However, in patients with cardiac tamponade, it is unclear how hemodynamic stability is maintained during the induction and maintenance of general anesthesia with remimazolam.

**Patient concerns:**

An 88-year-old male patient had developed hemopericardium due to penetration of a pigtail catheter into the left ventricle during pericardiocentesis, which was performed to treat massive pericardial effusion.

**Diagnoses:**

The patient was diagnosed with acute cardiac tamponade and a hemothorax. Hemopericardium and hemothorax were confirmed on chest radiography and computed tomography performed immediately after pericardiocentesis.

**Interventions:**

Decompressive pericardiostomy was performed through a left anterolateral thoracotomy with 1-lung ventilation under general anesthesia. Remimazolam was administered for total intravenous anesthesia.

**Outcomes:**

Severe hypotension and bradycardia occurred during the induction of anesthesia with remimazolam (6 mg/kg/hours).

**Lessons:**

Remimazolam may induce severe hemodynamic instability during induction of general anesthesia in patient with cardiac tamponade.

## 1. Introduction

Cardiac tamponade is a condition in which cardiac compression occurs due to the accumulation of fluid, blood, pus, gas, or tissue in the pericardial space. Acute cardiac tamponade is a life-threatening emergency that can lead to death; therefore, prompt and appropriate treatment is required. Pericardiocentesis is mainly used as a medical treatment for pericardial effusion; however, surgical treatment is required for acute cardiac tamponade caused by traumatic hemopericardium.^[1]^

Side effects, such as myocardial depression, systemic vasodilation, and decreased preload or heart rate, which may occur following general anesthesia, can cause cardiac collapse in cardiac tamponade, with the possibility of cardiogenic shock or arrest in severe cases. Therefore, it is important to determine the appropriate anesthetic method and agent for maintaining hemodynamic stability in patients with cardiac tamponade. Usually, ketamine, etomidate, and midazolam can be used with caution as induction agents for anesthesia.^[2]^

Remimazolam, a benzodiazepine drug, has recently been developed and is currently used for the induction and maintenance of anesthesia.^[3]^ Several studies have reported that the occurrence of hemodynamic depression is low, and remimazolam can be used for appropriate anesthesia in patients with severe cardiac dysfunction or as an anesthetic agent in cardiac surgery.^[4–7]^ Base on the report of hemodynamic stability of remimazolam, we decided to use remimazolam as an anesthetic agent for acute cardiac tamponade caused by pigtail catheter penetrating the left ventricle during pericardiocentesis for massive pericardial effusion. We report the experience of performing anesthesia with remimazolam in cardiac tamponade and the associated problems, along with a literature review.

## 2. Case presentation

The patients medical records were reviewed following ethical approval, and the requirement for informed consent was waived by the Institutional Review Board of the Presbyterian Medical Center (IRB no. E2022-056).

**Figure 1.**
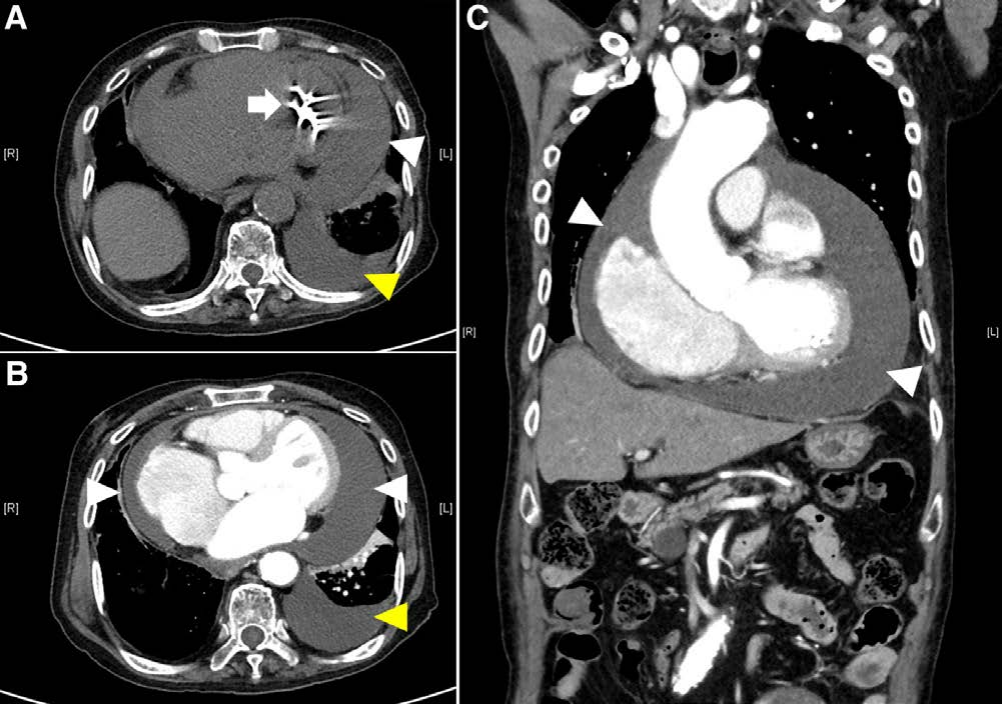
Computed tomography. Hemopericardium (white arrow head) and hemothorax (yellow arrow head) are observed. The pigtail catheter (white arrow) enters the left ventricle.

**Figure 2.**
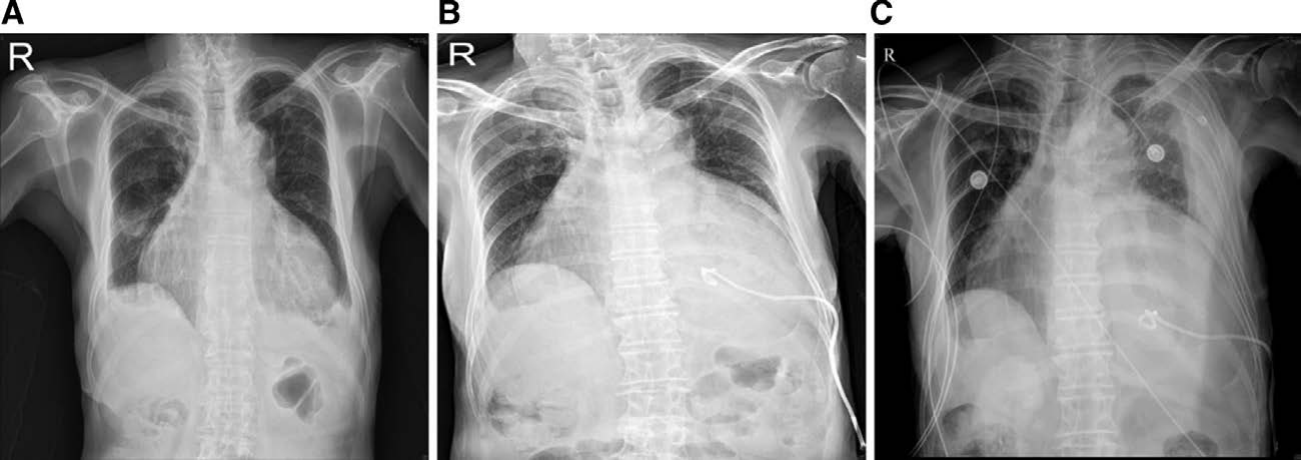
Chest radiography. (A) Before pericardiocentesis. (B) Immediately after pericardiocentesis. (C) Is a chest radiography at the time of the emergency room of our hospital, showing that the cardiac diameter is further increased, and the left lung is severe collapsed due to hemothorax.

An 88-year-old male (176 cm/58 kg) developed hemopericardium due to a pigtail catheter penetrating the left ventricle during pericardiocentesis, which was performed to treat massive pericardial effusion at another hospital. The patient was diagnosed with acute cardiac tamponade and was subsequently transferred to our hospital for emergency surgery. The patient had a medical history of atrial fibrillation and heart failure. Upon arrival at our emergency room, the patient complained of dyspnea and chest pain. Consciousness was alert and vital signs were relatively stable, blood pressure (BP) 110/75 mm Hg, heart rate (HR) 110 bpm, and oxygen saturation (SpO_2_) 99%. The laboratory results were in emergency room as follows: hemoglobin (Hb) 8.2 g/dL, hematocrit (Hct) 26.1%, platelet 248,000/μL, pro-B-type natriuretic peptide 3216 pg/mL (normal range: 0–526 pg/mL), and lactate 4.27 mmol/L (normal range: 0.5–2.2 mmol/L). The other laboratory values were within the normal ranges. Hemopericardium and hemothorax were confirmed on chest radiography and computed tomography performed immediately after pericardiocentesis at another hospital (Fig. 1). The hemopericardium and hemothorax severely deteriorated on chest radiography in the emergency room (Fig. 2). Atrial fibrillation with rapid ventricular response (112 bpm) was confirmed on electrocardiography. During the examination, the vital signs suddenly worsened with BP 72/27 mm Hg, HR 111 bpm, and SpO_2_ 96%. Oxygen was supplied via a facial mask. Colloid fluid loading, blood transfusion, and norepinephrine infusion were then initiated. Before transport to the operating room, vital signs were as follows: BP 84/55 mm Hg; HR 91 bpm; SpO_2_ 99%. The surgeon decided to proceed with the operation through left anterior thoracotomy. In addition, the operator noted the possibility of a cardiopulmonary bypass. Anesthesia was decided considering that the patient’s spontaneous breathing was unstable due to massive hemothorax, 1-lung ventilation was required to approach the lesion, and cardiopulmonary bypass was possible. Therefore, we planned to proceed with the operation after the induction of anesthesia with remimazolam and intubation using a double-lumen tube.

**Figure 3.**
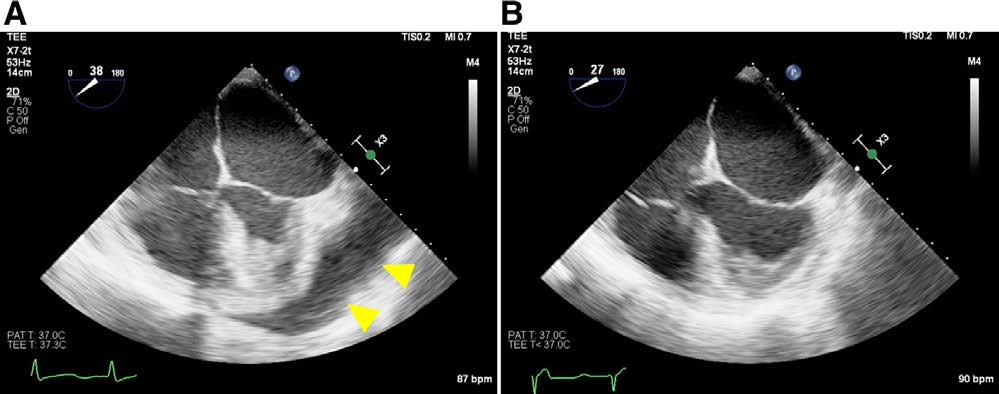
Transesophageal echocardiogram. (A) Before pericardial drainage, (B) after pericardial drainage. Hemopericardium (yellow arrow head) is completely removed after drainage.

**Figure 4.**
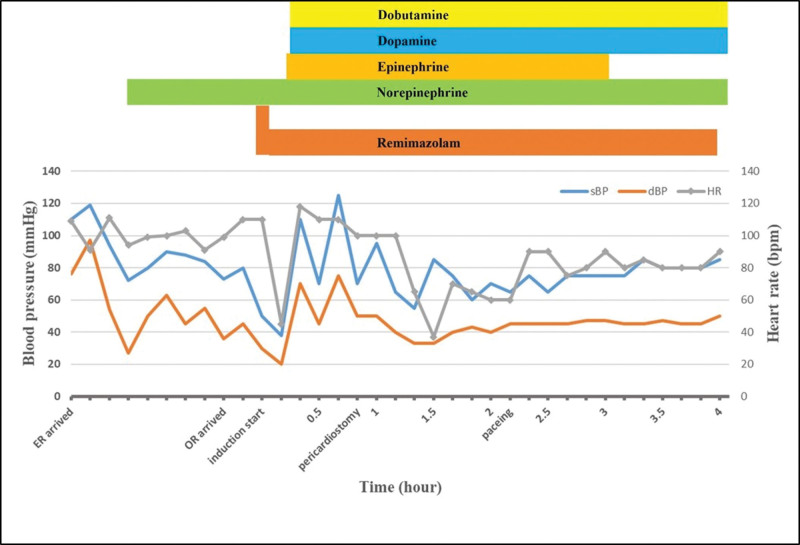
Perioperative hemodynamic changes. It shows an abrupt decrease in BP and HR after induction with remimazolam. Hemodynamic instability persists even after pericardiostomy has been performed. BP = blood pressure, dBP = diastolic blood pressure, ER = emergency room, HR = heart rate, OR = operating room, sBP = systolic blood pressure.

No premedications were administered. The following monitoring was conducted: noninvasive blood pressure, 5-lead electrocardiogram, pulse oximeter, Patient State Index, cerebral oximetry, and pulse CO-oximeter. Disposable defibrillator pads were attached. Upon arrival to the operating room, the patient’s vital signs were as follows: BP 73/36 mm Hg; HR 99 bpm; SpO_2_ 67%, and metal state was drowsy. preoxygenation was performed using 100% O2 via a facial mask. An arterial cannula was inserted into the radial artery to monitor the invasive BP, cardiac output/index, and stroke volume variation before induction. Anesthesia was induced while fluid resuscitation and norepinephrine infusion were maintained. For induction of anesthesia, remimazolam was administered intravenously at 6 mg/kg/hours. At this time, the BP and HR were 80/45 mm Hg and 110 bpm, respectively. Rocuronium 60 mg and fentanyl 50 μg were slowly administered intravenously after the patient lost consciousness, and remimazolam was adjusted to 1 mg/kg/hour. Manual ventilation was performed using a facial mask. The patient’s BP and HR suddenly decreased (BP 38/20 mm Hg, HR 45 bpm). It was judged as cardiogenic shock, and epinephrine 50 μg was immediately injected intravenously. BP and HR recovered to 125/75 mm Hg and 110 bpm after the additional use of epinephrine 25 μg. At this time, Hb 5.5 g/dL and Hct 18.2% were checked in the complete blood count test. Intubation was performed using a double-lumen tube, and transesophageal echocardiogram was placed (Fig. 3). A central venous catheter and pulmonary artery catheter (surgeon wanted) were inserted into the right internal jugular vein under ultrasound guidance. Epinephrine, norepinephrine, dopamine, and dobutamine were used for hemodynamic stabilization of the patient during surgery. The perioperative hemodynamic changes are shown in Figure 4.

Operation was started immediately after intubation. Decompression was performed by incision of the pericardium after approaching left anterolateral thoracotomy. The pigtail catheter had penetrated the apical portion of the heart. The catheter was removed, the cardiac opening was sutured, and pericardial and pleural hematoma was removed. A temporary pacemaker was placed to correct the hemodynamic instability, especially atrial fibrillation. The patient was transferred to the intensive care unit without recovering from anesthesia while maintaining vasopressor and inotropes. The patient recovered completely and was discharged approximately 10 weeks later.

## 3. Discussion

Cardiac tamponade is characterized by progressively smaller heart chambers and decreased myocardial diastolic compliance due to an increase in intrapericardial pressure, which result in limited cardiac filling and decreased stroke volume. Sympathetic and neurohumoral activation, such as increased heart rate, contractility, and vasoconstriction, occur compensatively for preserving cardiac output.^[1,2]^ Hemodynamic goals for cardiac tamponade can be summarized as “full (intravenous volume), fast (heart rate), and strong (myocardial contractility)”.^[2,8]^ However, general anesthesia can cause myocardial depression, vasodilation, and preload reduction. This can exacerbate hemodynamic compromise by dramatically reducing the compensation for patients with cardiac tamponade. In addition, positive pressure ventilation during general anesthesia can further reduce preload by reducing systemic venous return.^[1,2]^ The goal of general anesthesia for cardiac tamponade is to maintain hemodynamic stability until pericardial decompression. Therefore, selection of anesthetic methods and drugs for this purpose is important. Typically, anesthesia induction for surgical decompression of cardiac tamponade begins after the surgical team is ready for surgery, and spontaneous breathing must be maintained during surgery until decompression. In patients who cannot maintain spontaneous breathing or require positive pressure ventilation (PPV), it should be performed at low tidal volume, high respiratory rate, and low peak airway pressure. Ketamine is an anesthetic agent that increases BP and HR by increasing sympathetic activity, and is used in patients with hemodynamic instability.^[9]^ It is also useful when spontaneous respiration is required during surgery as the airway reflexes and respiratory drive are maintained.^[10]^ Therefore, ketamine is most often used for anesthesia in cardiac tamponade.

In this case, we used remimazolam for the induction and maintenance of anesthesia. Remimazolam is a recently developed benzodiazepine drug based on the molecular structure of midazolam and has a hemodynamic effect similar to that of midazolam. It has better hemodynamic stability than other intravenous anesthetic agents except ketamine.^[11,12]^ Also, it has less respiratory inhibition. however, unlike midazolam, it can be used for maintenance of anesthesia because it has a short context-sensitive half time.^[11]^

We initially planned to perform pericardiostomy under spontaneous breathing after induction with remimazolam and repair the left ventricular injury while maintaining anesthesia with remimazolam and fentanyl. However, 1-lung ventilation was required for the operation, and the patient’s spontaneous respiration was unstable due to worsening hemothorax; therefore, we modified the plan to perform intubation and then proceed with pericardial drainage. Remimazolam 6 mg/kg/hours was used for induction. Although induction was performed while maintaining fluid resuscitation and norepinephrine infusion, severe hypotension and bradycardia occurred. We deduce the cause as follows: First, it can be caused by the effects of remimazolam. This may be a result of the attenuation of the compensatory response to cardiac tamponade or direct myocardial depression and vasodilation by remimazolam. Second, it can be exacerbated by PPV because positive thoracic pressure can further impair cardiac filling in cardiac tamponade. When a neuromuscular blocking agent was injected and PPV via a facial mask was performed, sudden hemodynamic changes occurred. The aggravation of hypotension and the abrupt decrease in HR may be attributed to the decrease in preload caused by PPV. Third, when hemodynamic instability worsened, Hb 5.5 g/dL and Hct 18.2% on complete blood count were significantly reduced compared to preoperative tests (Hb 8.2 g/dL, Hct 26.1%). This finding suggests the presence of persistent bleeding and subsequent hypovolemia. Hypovolemia may have further reduced the preload. Fourth, it may have been caused by the patient’s underlying disease, atrial fibrillation, or heart failure. The fact that hemodynamic compromise was maintained even after pericardial decompression suggests the possibility of exacerbation by hypovolemia, atrial fibrillation, and heart failure. We believe that all of these causes contributed to the occurrence of severe hemodynamic instability in this case.

The sympathetic effects of intravenous anesthetic agents are poorly understood. Ketamine increases central sympathetic activity and consequently increases BP and HR.^[9,10]^ Although this is generally a disadvantage for anesthesia induction, ketamine is the most suitable drug along with etomidate and midazolam in hemodynamically unstable patients such as those with cardiac tamponade. However, ketamine can also induce hypotension owing to direct myocardial depression in the absence of adequate sympathetic and myocardial reserves. In this case, hemodynamic instability may have deteriorated even with ketamine, because the patient had atrial fibrillation and heart failure. The effects of remimazolam on the sympathetic nervous system are unknown, and further investigation is needed.

## 4. Conclusion

Remimazolam has better hemodynamic stability than other intravenous anesthetic agents. However, its use as an anesthetic agent in hemodynamically unstable cardiac tamponade may induce severe cardiovascular collapse. Further studies on the use of remimazolam in cardiac tamponade should be conducted.

## Author contributions

**Conceptualization:** Yu Yil Kim.

**Data curation:** Geonbo Kim, Junyoung Park.

**Investigation:** Hyun Joo Heo.

**Supervision:** Yu Yil Kim.

**Writing – original draft:** Hyun Joo Heo, Geonbo Kim.

**Writing – review & editing:** Hyun Joo Heo, Yu Yil Kim.
